# Books and Magazines

**Published:** 1904-12

**Authors:** 


### Books and Magazines.

ESSENTIALS OF NERVOUS DISEASES AND INSANITY.—Their Symptoms and Treatment. By John C. Shaw, M. D., late Clinical Professor of Diseases of the Mind and Nervous System, Long Island College Hospital Medical School. Fourth edition, thoroughly revised. By Smith Ely Jelliffee, Ph. G., M. D., Clinical Assistant, Columbia University, Department of Neurology; Visiting Neurologist, City Hospital, New York. 12mo volume of 196 pages, fully illustrated. Philadelphia, New York, London: W. B. Saunders & Company. 1904. Cloth, $1.00 net.

Of the progress made in every branch of medicine during the last few years, none has been more prominent than that considering diseases of the nervous system and of the mind. Dr. Smith Ely Jelliffe, therefore, in making the revision for this new fourth edition, has found it necessary to recast the work entirely, bringing the order of arrangement in accord with the present knowledge of these important subjects. Quite a commendable change in arrangement is the grouping of subjects in such a way as to bring out the natural relations of affiliated nervous disorders. This will be found of great service to the student.

In the section on disorders of the mind, the general views of such leading psychologists as Ziehen, Weygandt, Kaepelin, Berkeley, and Peterson have been carefully weighed. This new fourth edition is well worthy our recommendation, and we give it most heartily.

ESSENTIALS OF BACTERIOLOGY. By M. V. Ball, M. D., formerly Resident Physician at the German Hospital, Philadelphia. Fifth edition, thoroughly revised. By Karl M. Vogel, M. D., Assistant Pathologist at the College of Physicians and Surgeons (Columbia University), New York City. 12mo volume of 343 pages, with 96 illustrations, some in colors, and six plates. Philadelphia, New York, London: W. B. Saunders & Company. 1904. Cloth, $1.00 net.

Within the last few years rapid progress in bacteriology has involved many radical changes in the science, necessitating a thorough revision in the preparation of this edition. It is with pleasure we note the insertion of all the recent advances in the subjects of Immunity, Tuberculosis, Yellow Fever, Dysentery, Bubonic Plague, and other infectious diseases, making the work reflect as faithfully as possible the present status of Bacteriology. We can confidently say that this book in the present edition will be found of inestimable service to the student.

Essentials of Materia Medica and Prescription Writing. By Henry Morris, M. D., College of Physicians, Philadelphia. Sixth edition, thoroughly revised. By W. A. Bastedo, Ph. G., M. D., Tutor of Materia Medica and Pharmacology at the Columbia University (College of Physicians and Surgeons), New York City. 12mo volume of 295 pages. Philadelphia, New York, London: W. B. Saunders & Company. 1904. Cloth, $1.00 net.

Dr. Bastedo, in making the revision of Dr. Morris' "Essentials of Materia Medica," has furnished the student with a work complete and up to date in every particular. Much of the text has been in great part rewritten. There have been introduced articles on adrenalin, stypticin, and on the iodine and silver synthetics. The present sixth edition is all that could be desired.

